# *Lactobacillus* isolates from healthy volunteers exert immunomodulatory effects on activated peripheral blood mononuclear cells

**DOI:** 10.7555/JBR.27.20120074

**Published:** 2012-12-24

**Authors:** Keyi Sun, Chao Xie, Donghua Xu, Xiaofan Yang, James Tang, Xiaohui Ji

**Affiliations:** a Department of Microbiology and Immunology, Nanjing Medical University, Nanjing, Jiangsu 210029, China;; b Cultech Limited, Unit 2 Christchurch Road, Baglan Industrial Park, Port Talbot, SA12 7BZ, Wales, UK.

**Keywords:** lactobacilli, probiotics, Th1, Th2, cytokine, immuno-modulation

## Abstract

As probiotics in the gut, Lactobacilli are believed to play important roles in the development and maintenance of both the mucosal and systemic immune system of the host. This study was aimed to investigate the immuno-modulatory function of candiate lactobacilli on T cells. Lactobacilli were isolated from healthy human feces and the microbiological characteristics were identified by API 50 CHL and randomly amplified polymorphic DNA (RAPD) assays. Anti-CD3 antibody activated peripheral blood mononuclear cells (PBMCs) were treated by viable, heat-killed lactobacilli and genomic DNA of lactobacilli, and cytokine profiles were tested by ELISA. Isolated lactobacilli C44 and C48 were identified as *L. acidophilus* and *L. paracacei*, which have properties of acid and bile tolerance and inhibitor effects on pathogens. Viable and heat-killed C44 and C48 induced low levels of proinflammatory cytokines (TNF-α, IL-6 and IL-8) and high levels of IFN-γ and IL-12p70 in PBMCs. In anti-CD3 antibody activated PBMCs, viable and heat-killed C44 increased Th2 cytokine levels (IL-5, IL-6 and IL-10), and simultaneously enhanced Th1 responses by inducing IFN-γ and IL-12p70 production. Different from that of lactabacillus strains, their genomic DNA induced low levels of IL-12p70, IFN-γ and proinflammatory cytokines in PBMCs with or without anti-CD3 antibody activation. These results provided in vitro evidence that the genomic DNA of strains of C44 and C48, especially C44, induced weaker inflammation, and may be potentially applied for treating allergic diseases.

## INTRODUCTION

Lactobacilli are the major members of probiotics, which are defined by the FAO/WHO as “live microorganisms which when administered in adequate amounts confer a health benefit on the host”[1]. Members of these genera are commensal bacteria in human intestine and have a long history of safe uses. It is well documented that various lactobacillus species contribute to the health of the host, which is known to modulate immune responses[2]. The most interesting thing is their property in regulating the polarization of naive immune system by skewing it away from T helper 2 (Th2) toward Th1 responses, and thus promoting cell mediated immunity[3], which will lead to the application in prevention and treatment of allergic diseases.

There has been a significant increase in the prevalence of allergic diseases over the past 2 to 3 decades such as atopic dermatitis, atopic eczema, and allergic rhinitis. Among factors possibly contributing to the increase in the prevalence of allergic diseases, modification of the intestinal flora or lack of microbial exposure during childhood has been proposed. Th2-cytokines increase the production of IgE and stimulate mast cells and eosinophils, whereas Th1-cytokines, such as interferon (IFN)-γ, may suppress IgE synthesis and stimulate the expression of secretory IgA[4]–[6]. Although there were substantial in vivo evidence from animal models and clinical trials on Th2 cytokine inhibitory effects of lactobacilli[3],[7],[8] and in vitro evidence that lactobacilli stimulated antigen presenting cells (APC) that trigger T cell polarization[9],[10], few direct cellular witnesses from T cells have been provided in vitro.

As probiotics, a candidate lactobacillus should survive passage through the gastrointestinal tract and transiently colonize the host epithelium[1]. The most important property for survival is the tolerance of highly acidic conditions present in the stomach and the concentrations of bile salts in the small intestine. Besides, probiotic lactobacilli are able to inhibit, displace and compete with pathogens, and enhance mucosal barrier activity, although these abilities are strain-dependent.

In the present study, two lactobacillus strains, *L. acidophilus* and *L. paracasei*, were selected from bacteria isolated from healthy volunteers to determine the effects of lactobacilli on T cell polarization in vitro, including the capacity to induce immune responses in peripheral blood mononuclear cells (PBMCs).

## MATERIALS AND METHODS

### Isolation and biochemical characterization of candidate lactobacilli

Fecal samples were provided by two healthy volunteers who did not take any probiotic-based supplements. A 10^-1^ dilution was prepared by sterile Maximal Recovery Diluent (MRD, Oxoid UK) and serial dilutions to 10^-7^ were generated. Dilutions including 10^-4^, 10^-5^, 10^-6^ and 10^-7^ were plated onto Man-Rogosa-Sharpe (MRS) agars (Oxoid, Basingstoke, Hampshire, UK) using modified Miles & Misra plating technique (10×10 µL) and allowed to dry and then were incubated anaerobically at 37°C for 72 hours[13]. All presumptive lactobacillus colonies were subcultured onto MRS agar (Oxoid) and incubated anaerobically at 37°C for 48 hours. All catalase negative, Gram positive bacilli were identified. For experimental use, strains were cultured anaerobically at 37°C in MRS broth (Oxoid) to early stationary phase, using three successive subcultures (1% v/v inoculation; 12-15 h). Carbohydrate fermentation profile was obtained by API 50 CHL tests according to the manufacturer specification (BioMérueux, France). The Apiweb^R^ identification software was used to interprete carbohydrate fermentation results. *L. acidophilus* and *L. paracasei* were selected because they are the major lactobacillus strains and may have potential immunoregulatory effects on T cell polarization by inducing IL-12 and IFN-γ secretion from dendritic cells (DC)[11] and macrophages[12].

### Molecular characterization of candidate lactobacilli

Genomic DNA of candidate lactobacilli were extracted using Bacterial Genomic DNA Kit (Sigma, St. Louis, MO), overnight cultured bacteria were lysed by lysis solution at 55°C for 10 minutes, mixed with ethanol and then centrifuged at 6,500 g for 1 minute, washed and finally eluted with elute solution. DNA genotypes were analyzed using the randomly amplified polymorphic DNA (RAPD) fingerprinting method[14] with RAPD Ready-to-Go beads (GE Healthcare, UK) and random primer (MWG Biotech, Germany) at a final volume of 25 µL, including 2.5 µL of PCR buffer, 5 µL of Q-solution, 2 µL of 25 mmol/L MgCl_2_, 2 µL of 10 mmol/L dNTPs mixture, 1 µL of primer, 1 µL of template DNA and 1 unit of *Taq* DNA polymerase. The PCR was run for 5 minutes at 94°C, 5 minutes at 36°C and 5 minutes at 72°C, 35 cycles of 94°C for1 minute, 36°C for 1 minute, 72°C for 2 minutes, and a final extension of 72°C for 6 minutes. Standard strains of lactobacillus were used as positive controls.

### Bile and acid tolerance assays

Tolerance to bile was assessed by investigating the ability of strains to grow in the presence of different concentrations of bovine bile (Oxiod), as previously described [15]. Freshly cultured lactobacilli (final concentration at 10^6^cell/mL) were inoculated in MRS broth containing 0, 0.3%, 1.0%, 2.0%, and 3.0% (w/v) bovine bile and incubated anaerobically at 37°C. Bacterial growth was monitored on MRS agar (Oxoid) by viable count every 1 hour for 5 hours. Acid tolerance assay of freshly cultured lactobacilli (at a final concentration of 10^6^ cell/mL) was performed in MRS broth at different pH values of 2.0, 3.0, 4.0 and incubated anaerobically at 37°C[16]. Bacterial growth was monitored on MRS agar (Oxoid) by viable count at 1, 2 and 3 hours. Three independent experiments were carried out in triplicate.

### Pathogen inhibition experiments

Cell-free supernatant from overnight cultured lactobacilli was obtained by centrifugation at 2000 rpm for 10 min and filtered to remove the bacteria, then divided into two groups: the high pH group neutralized with NaOH and the low pH group (without neutralization). Normal MRS broth was used as a control. Different pathogens were cultured in LB broth including C2 (*E. coli*), C5 (*S. typhimurium*), C11 (*E. faecalis*), C14 (*K. pneumoniae*), C25 (*S. flexneri*) and QC8 (*P. aeruginosa*) at 37°C overnight and re-cultured in the two groups mentioned above at 37°C and viable count was performed at 5 hours and 24 hours on LB agar. Increasing or reducing percentage of pathogens were calculated using the following formula: (viable bacteria in the high pH group or in the low pH group-viable bacteria in normal MRS group)/viable bacteria in normal MRS group×100%.

### Preparation of stimulus

Lactobacilli were cultured overnight at 37°C in MRS broth, collected by centrifugation, and washed several times with sterile PBS and diluted in RPMI 1640 medium as viable lactobacilli; viable bacteria were killed by heating at 60°C for 1 hour and viable count was carried out to make sure no viable bacteria survived. Bacteria were stored at -80°C as heat-killed lactobacilli. Genomic DNA of lactobacilli was extracted as described before.

### PBMCs isolation

PBMCs were isolated from peripheral blood of healthy donors. Briefly, after a Histopaque 1077 (Sigma) gradient centrifugation, mononuclear cells were collected, washed and adjusted to 1×10^6^ cells/mL in RPMI 1640 medium supplemented with 10% fetal bovine serum (Sigma).

### Cytokine production from PBMCs

PBMCs (2×10^5^ cells/mL) were plated in duplicate in a 96-well culture plate and stimulated with overnight cultured viable lactobacilli at a multiple of infection (MOI) of 1 or heat-killed lactobacilli at an MOI of 10 or bacterial genonomic DNA (3 µg/mL) in 200 µL RPMI 1640. C11 (*E. faecalis*) was used as a Gram-positive opportunistic pathogenic bacteria control. C30 (*B. bifidum*), the most traditionally used microorganism as probiotic, was selected as a probiotic control because of its promotion of Th1 polarization[17]. LPS (10 µg/mL, Sigma) as an inflammatory positive control, and unstimulated PBMCs were used as a negative control. PBMCs were also activated with anti-CD3 antibody (5 µg/mL) and stimulated with viable lactobacilli or heat-killed lactobacilli or genomic DNA of both stains. After 24 or 72 hours of stimulation at 37°C in an atmosphere of air with 5% CO_2_, the supernatants were collected, clarified by centrifugation and stored at -20°C until cytokine analysis.

### Cytokine determination by ELISA

Cytokine concentrations in culture supernatants were assayed by sandwich ELISA using BD kits (BD Biosciences, USA) for tumor necrosis factor (TNF)-α, interleukin (IL)-10, IL-6, IL-8, IFN-γ, IL-12p70 and transforming growth factor (TGF)-β in the supernatant of cells stimulated for 24 hours and IL-4, IL-5, IL-13 in the supernatant of cells stimulated for 72 hours according to the manufacturer's recommendations.

### Statistical analysis

All the data were expressed as mean±SD. Comparison between groups were made using one-way analysis of variance (one-way ANOVA) and Student's *t* test. Differences were considered statistically significant for *P* value < 0.05.

## RESULTS

### Categorization and microbiological characters of potential probiotic lactobacilli

**Fig. 1:**
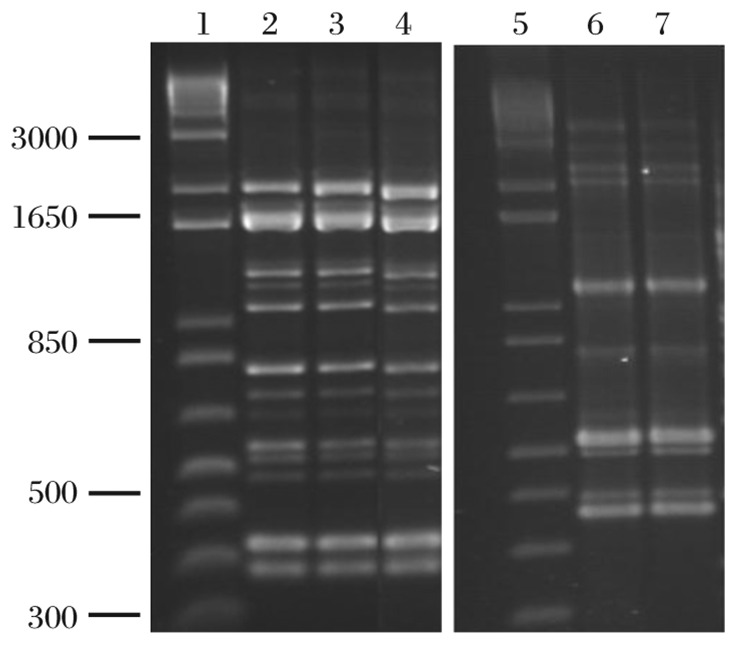
Detection of C44 and C48 by RAPD fingerprints. PCR fingerprinting Lactobacillus strains (with random primer) to cluster identical isolates and the standard strains are shown. Lane 1 and 5: Marker; lane 2 and lane 3: C44; lane 4: LMG9433; lane 6: C48; lane 7: LMG7955. LMG9433 is a standard train of *L. acidophilus* and LMG7955 is a standard strain of *L. paracasie.*

Colonies of presumptive lactobacilli were small, circular with a smooth edge, and convex with a glistening translucent appearance. Gram staining showed slender Gram-positive rods in single cells. On the basis of carbohydrate utilization profiles of the isolates, two strains among the isolated colonies were selected, named and identified: C44, *L. acidophilus*, the degree of identity (ID) was 94.5%; C48, *L.* paracacei, the ID was 95.5%. RAPD analysis showed that the strains had the same fingerprints as the standard strains (***Fig. 1***).

### Tolerance to bile salt and low pH

The two isolated strains survived in either acidified MRS broth or MRS broth with bile salt (***Table 1*** and ***Table 2***). After 3 hours of exposure, C44 and C48 survived at pH 2.0 and even grew in MRS broth with higher pH (pH 3.0, pH 4.0). After 5 hours of exposure, both isolates survived in 3% bile salt and multiplied in lower concentrations (2%, 1%, and 0.03%).

**Table 1:** Tolerance of *lactobacilli* to different bile concentrations (×10^6^ CFU/mL) (*n* = 3)

Bile concentration	*Lactobacillus* strains	Incubation (hours)					
		0	1	2	3	4	5
0	C44	6.0±0.7	8.5±1.0	9.4±0.7	10.0±1.9	43.0±3.7	76.0±6.5
	C48	7.0±0.3	8.0±0.8	20.0±1.2	60.0±5.3	80.0±8.2	105.0±11.2
0.30%	C44	6.0±0.6	6.0±0.5	3.5±0.4	6.3±0.7	9.0±1.1	11.0±1.2
	C48	7.0±0.4	7.8±0.5	11.0±0.8	12.0±1.2	57.0±5.3	58.0±4.5
1.0%	C44	6.0±0.4	3.5±0.3	5.8±0.3	5.0±0.4	6.3±0.8	7.6±0.8
	C48	7.0±0.4	6.5±0.4	6.6±0.7	6.8±1.1	10.6±1.4	10.8±0.8
2.0%	C44	6.0±0.5	2.0±0.4	2.4±0.4	5.4±0.4	6.2±0.7	7.0±0.7
	C48	7.0±0.6	6.3±0.3	6.3±1.1	6.4±0.6	8.0±1.2	8.4±1.1
3.0%	C44	6.0±0.8	3.0±0.5	5.5±0.2	4.8±0.3	4.5±0.4	6.3±0.5
	C48	7.0±0.3	6.0±0.6	6.1±1.2	6.1±0.8	6.1±0.5	7.5±0.5

Lactobacilli were cultured in RPMI 1640 at initial concentrations of 10^7^, 10^6^ and 10^5^ CFU/mL with or without antibiotics. Data of viable count are expressed as mean ±SD for *n* = 3.

**Table 2:** Tolerance of C44 and C48 to different pH values (×10^6^ CFU/mL) (*n* = 3)

pH	*Lactobacillus* strains	Incubation (hours)			
		0	1	2	3
0	C44	6.0±0.7	8.5±1.0	9.4±0.7	10.0±1.9
	C48	7.0±0.3	8.0±0.8	20.0±1.2	60.0±5.3
2.0	C44	6.0±0.6	6.0±0.5	3.5±0.4	6.3±0.7
	C48	7.0±0.4	7.8±0.5	11.0±0.8	12.0±1.2
3.0	C44	6.0±0.4	3.5±0.3	5.8±0.3	5.0±0.4
	C48	7.0±0.4	6.5±0.4	6.6±0.7	6.8±1.1
4.0	C44	6.0±0.5	2.0±0.4	2.4±0.4	5.4±0.4
	C48	7.0±0.6	6.3±0.3	6.3±1.1	6.4±0.6

C44 and C48 were cultured in MRS at different pH values of 2.0, 3.0 and 4.0. Viable count was carried out at 1, 2 and 3 hours. Values are mean ±SD for *n* = 3.

### Inhibitory effects of C44 and C48 on pathogenic bacteria

**Fig. 2:**
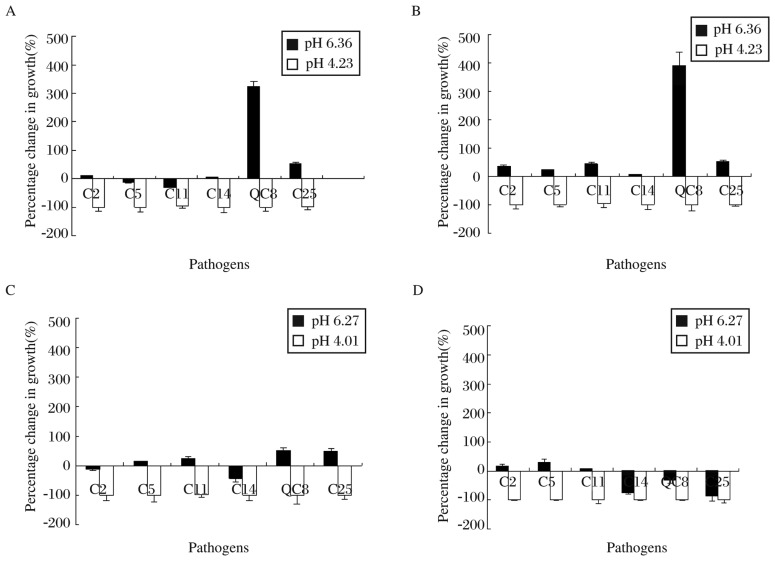
Inhibition of pathogen growth by lactobacilli-free culture medium. Pathogens were cultured in neutralized and non-neutralized supernatants of Lactobacillus strains C44 or C48 for 5 and 24 hours, respectively. Viable count was performed and changes in percentage of growth were determined. A and B: pathogens cultured in supernatant of C44 for 5 and 24 hours, respectively; C and D: pathogens cultured in supernatant of C48 for 5 and 24 hours, respectively. Pathogens were: C2 (*E. coli*), C5 (*S. typhimurium*), C11 (E. *faecalis*), C14 (*K. pneumoniae*), C25 (*S. flexneri*) and QC8 (*P. aeruginosa*). Values are mean±SD for *n* = 3.

All pathogens (*E. coli, S. typhimurium, E. faecalis, K. pneumoniae, S. Flexneri* and *P. aeruginosa*) were inhibited after incubation for 5 and 24 hours in culture medium of C44 and C48 under low pH (***Fig. 2***), respectively. But in NaOH neutralized culture medium of C44, most pathogens were inhibited except *P. aeruginosa* and *S. flexneri* after 5 hours of culture. The same results were obtained after 24 hours of culture except a slight increase in the number of *E. coli, S. typhimurium* and *E. faecalis*. In neutralized MRS broth of C48, the number of pathogens slightly increased (increase≤50%) after 5 hours of culture, but after 24 hours of culture, most pathogens were inhibited except *E. coli* and *S. typhimurium*.

### Effects of lactobacillus concentrations on the secretion of IL-10 by PBMCs with and without antibiotics

To determine the viability of lactobacilli in RPMI 1640 was influenced by antibiotics, we grew C44 and C48 in different concentrations in RPMI 1640 with or without antibiotics. The results revealed that C44 and C48 grew at high concentration (10^7^ CFU/mL) but did not grow at lower concentrations (10^6^ CFU/mL and 10^5^ CFU/mL) in RPMI 1640 in the absence of antibiotics. However, in the presence of antibiotics, lactobacilli at all concentrations died within 4 hours (***Table 3***). The results illustrated that the effects of viable lactobacilli were abolished in the presence of antibiotics in culture medium.

**Table 3:** Growth behavior in RPMI 1640 with or without antibiotics (×10^5^CFU/mL) (*n*=3)

Bacteria	Antibiotics	Incubation (hours)					
		0	4	6	8	10	24
C11	-	380±35	1,700±206	1,900±200	2,000±208	2,600±330	3,400±508
	+	380±35	0.22±0.10	0.16±0.05	0	0	0
	-	38±4	1,500±110	2,300±306	2,400±310	2,700±302	3,100±350
	+	0 38±4.20	0.02±0.01	0	0	0	0
	-	3.80±0.20	11±4.20	20±2.40	25±3.10	50±6	150±11
	+	3.80±0.30	0	0	0	0	0
C44	-	480±22	1.48±0.80	23±3.20	46±5.20	11.4±2.70	400±35
	+	480±23	0	0	0	0	0
	-	0 48±2.50	0.13±0.07	7.40±0.85	2.40±0.30	16±2.10	60±11
	+	48±20	0	0	0	0	0
	-	4.8±0.20	0.12±0.03	0.32±0.10	0.78±0.11	0.77±0.09	0.65±0.08
	+	4.8±0.20	0	0	0	0	0
C48	-	90±12	122±14.30	266±30.80	400±45.2	5800±62	8000±100
	+	90±15	0	0		0	0
	-	0 9±1.80	45±5.70	26±4.20	20±3.10	27±3.20	38±3.50
	+	0 9±1.80	0	0	0	0	0
	-	0 1±0.20	0.16±0.04	0.74±0.07	1.24±0.21	1.80±0.18	3.80±0.45
	+	0 1±0.40	0	0	0	0	0

C44 and C48 were cultured in MRS broth containing 0, 0.3%, 1.0%, 2.0% and 3.0% bovine bile, respectively. Viable count was carried out every 1 for 5 hours. Values are means±SD for *n* = 3. C11, *E. faecalis*; C44 and C48: *Lactobacillus* strains.

**Fig. 3:**
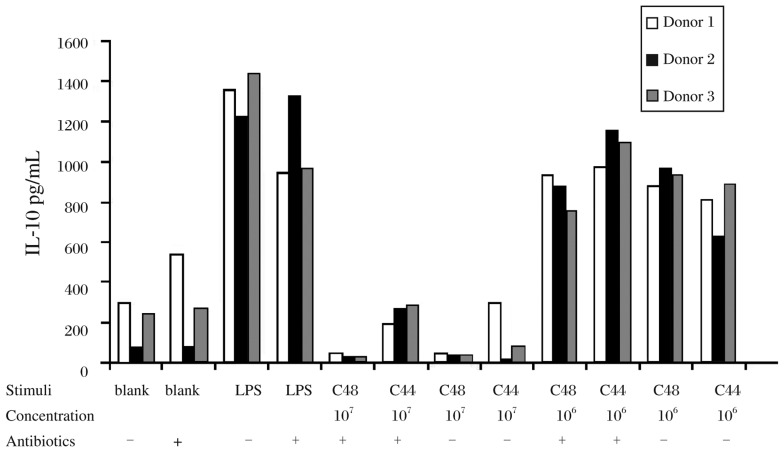
IL-10 production from PBMCs stimulated with lactobacilli in different concentrations with or without antibiotics. IL-10 secretion of PBMCs from 3 donors stimulated with viable C44 and C48 at 10^6^ and 10^7^ CFU/mL in the presence or absence of antibiotics.

To determine if living lactobacilli influence cytokine production in human PBMCs, we stimulated cells with different doses of living lactobacilli (at an MOI of 1 and 10) in RPMI 1640 with or without antibiotics for 24 hours. As shown in ***Fig. 3***, all lactobacilli at 10^7^ CFU/mL and 10^6^ CFU/mL induced low level of IL-10 with or without antibiotics. All the results indicated that viable C44 and C48 at 10^6^ CFU/mL had no influence on cytokine production of PBMCs. In view of this, PBMCs stimulated with viable lactobacilli at 10^6^ CFU/mL without antibiotics were applied in the subsequent experiments.

### Profiles of cytokine secretion in PBMCs stimulated with viable and heat-killed lactobacilli and their DNA

**Fig. 4:**
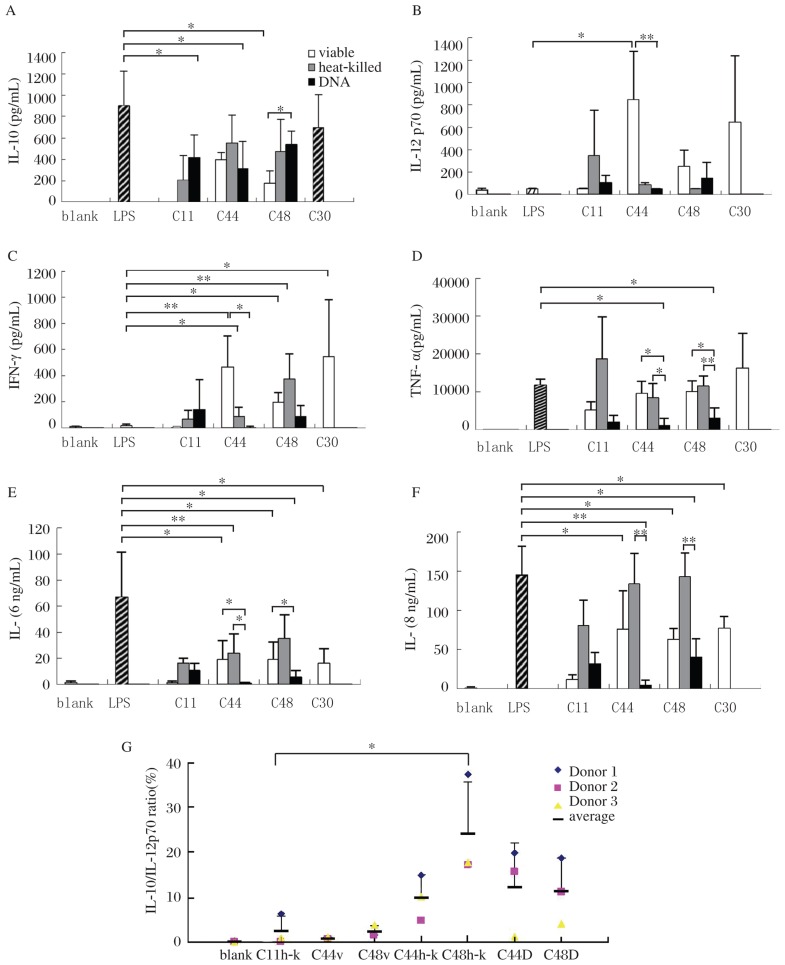
Profiles of cytokines produced by PBMCs induced by different stimuli from bacteria. Cytokine response after 24 hours of culture with various stimuli of bacteria are shown in A. Ratios of IL-10/IL-12p70 are shown in B. C11 (*E. faecalis*) and C30 ( *B. Bifidum*) are controls of opportunistic pathogens and probiotics (v:viable, h-k: heat-killed and D: DNA). Values are mean ± SD for *n*=3, *P < 0.05, **P < 0.01.

As shown in ***Fig. 4***, viable and heat-killed C44 and C48 induced the secretion of IL-12p70 and IFN-γ, which are critical for promoting cell immunity. Specifically, viable C44 significantly induced IL-12p70 (*P* < 0.05). Heat-killed C44 and viable C48 had remarkably potent activities on IFN-γ production (*P* < 0.05), while viable C44 and heat-killed C48 had even more potent effects (*P* < 0.01). On the contrary, genomic DNA of both lactobacilli did not induce the secretion of IFN-γ and IL-12p70.

TNF-α, IL-6 and IL-8, considered as proinflammatory cytokines, were induced by the lactobacilli, although they were lower than those induced by LPS in PBMCs. In general, DNA of both lactobacilli induced significantly lower TNF-α, IL-6 and IL-8 secretion than LPS did (*P* < 0.05). Viable C44 and C48 induced lower levels of IL-6 and IL-8 (*P* < 0.05) compared with LPS.

IL-10 and TGF-β are important anti-inflammatory cytokines to suppress cell immunity. Viable C44 and its DNA, as well as viable C48 induced lower IL-10 levels than LPS did (*P* < 0.05). But no obvious effects were observed on TGF-β production by both lactobacilli (data not shown).

As IL-10 and IL-12 appeared to be the most discriminative cytokines, IL-10/IL-12 ratio was used to distinguish between strains exhibiting a “pro-“ vs “anti-inflammatory” profile[18] as shown in ***Fig. 4G***. Heat-killed lactobacilli and their DNA evoked high IL-10/IL-12p70 ratio, and the notable inducer was heat-killed C44 (*P* < 0.05).

In general, C44 induced low levels of proinflammatory and anti-inflammatory cytokines and a high ratio of IL-10/IL-12. This indicated that C44 have the potentiality to anti-inflammation.

### Profiles of cytokine secretion in anti-CD3 antibody activated PBMCs stimulated with viable and heat-killed Lactobacilli and their genomic DNA

**Fig. 5:**
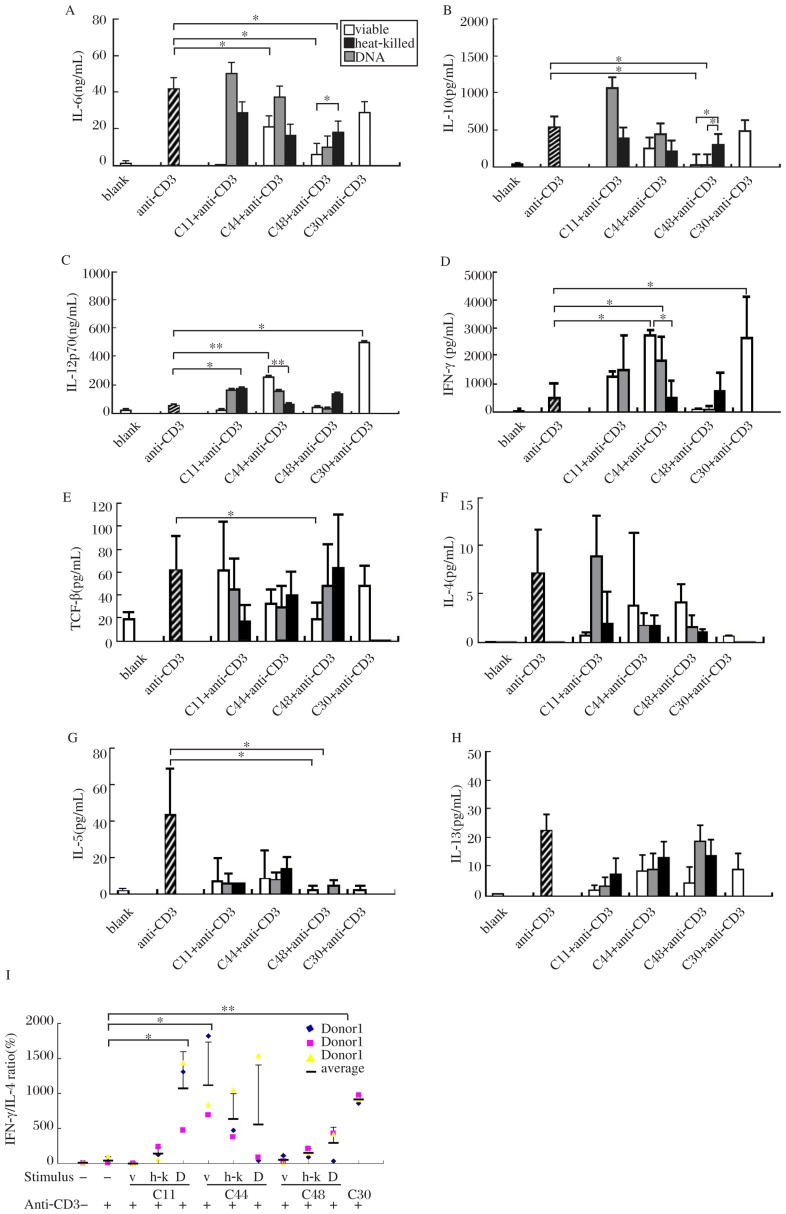
Profiles of cytokines from anti-CD3 activated PBMCs induced by different stimuli from bacteria. TCF-β (E), IL-10 (B), IL-6 (A), IL-8, IFN-γ (D) and IL-12p70 (C) response after 24 hours of culture, and IL-4 (F), IL-5 (G), IL-13 (H) response after 72 hours of culture with various stimuli together with anti-CD3 antibody are shown in (A). Ratios of IL-10/IL-12p70 are shown in *Fig. I.* C11 (*E. faecalis*) and C30 (*B. Bifidum*) are controls of opportunistic pathogens and probiotics (viable, heat-killed and DNA of bacteria). Values are mean±SD for *n*=3. Significance levels are indicated and denoted as *P < 0.05, **P < 0.01.

In an attempt to investigate whether lactobacilli regulated T cell polarization, we activated T cells in PBMCs by anti-CD3 antibody and then stimulated these cells with viable, heat-killed and genomic DNA of C44 and C48. IFN-γ and IL-12p70 are the key cytokines to promote naive T cells to differentiate into Th1 cells[19],[20]. Our results showed that IFN-γ secretion was promoted by viable, heat-killed C44 (*P* < 0.05); similarly, IL-12p70 was triggered by viable C44 (*P* < 0.01). But genomic DNA of C44 and C48 failed to induce IFN-γ and IL-12p70 production. Produciton of Th2 cytokines (IL-4, IL-5, IL-6, IL-10 and IL-13), was suppressed by C44 and C48[21]. Especially, viable and heat-killed C48 significantly suppressed the secretion of IL-5, IL-6 and IL-10 induced by anti-CD3 antibody (*P* < 0.05). But genomic DNA of C48 and C44 failed to do so. As a suppressive factor, TGF-β secretion remained invariable when anti-CD3 antibody activated PBMCs were treated with C44 and C48. Only viable C48 can downregulate the production of TGF-β (***Fig. 5A-H***). In brief, lactobacilli C44 can skew T cell polarization toward Th1 via enhancing the production of Th1 cytokines (IFN-γ and IL-12p70) as well as inhibiting Th2 cytokine (IL-5, IL-6 and IL-10) secretion.

Th1/Th2 balance is commonly monitored by IFN-γ/IL-4 ratio because IFN-γ and IL-4 are classical antagonistic signature cytokines for Th1 and Th2 activity. In this study, among the stimulus, only viable and heat-killed C44 induced high ratio of IFN-γ/IL-4 (*P* < 0.05) in anti-CD3 activated PBMCs. As controls, C11 (a opportunistic pathogen[22]) induced low ratio, while C30 (Bifidobacteria) induced obviously high ratio of IFN-γ/IL-4 (*P* < 0.01)(***Fig. 5I***).

### Differences in their effects on cytokine production between viable, heat-killed and their genomic DNA of lactobacilli

Viable and heat-killed lactobacilli were similar in their effects on cytokine secretion, while apparent differences were observed between bacteria and their genomic DNA. C44 DNA induced low levels of IL-12p70 (*P* < 0.01) and IFN-γ (*P* < 0.05), both from PBMCs (***Fig. 4***) and anti-CD3 antibody activated PBMCs compared with viable C44 (***Fig. 5***). On the other hand, DNA induced low levels of proinflammatory cytokines, including IL-6, IL-8 and TNF-α in PBMCs (***Fig. 4***). DNA of C44 triggered lower levels of IL-6 than viable C44 (*P* < 0.05) and heat-killed C44 (*P* < 0.05), and simultaneously induced lower levels of IL-8 than heat-killed C44 (*P* < 0.01). Similar to C44, DNA of C48 evoked lower levels of IL-6 (*P* < 0.05) and IL-8 (*P* < 0.01) than heat-killed C48, and similar results were obtained on TNF-α production upon stimulation by viable C48 (*P* < 0.05) and heat-killed C48 (*P* < 0.01). These results indicated that DNA of lactobacilli, especially of C44, induced weaker cellular immunity, which leads to inflammation than bacteria itself.

## DISCUSSION

To elucidate the effects of candidate probiotic lactobacilli on immuno-regulation, especially on T cell polarization, we isolated C44 and C48, identified to be *L. acidophilus* and *L. paracacei*, from healthy human feces. They have the probiotic properties including acid and bile tolerance, which allow them to survive and enter the intestine, and inhibition of some pathogens. Thus, C44 and C48 are able to localize in the digestive tract and restore intestinal homeostasis, thus improving mucosal barrier functions.

For the widely known strain-specific ability of lactobacilli to modulate the immune responses, it is necessary to investigate the characteristics of candidate stains for their potential therapeutic applications. In Pochard's study, lactobacillus-exposed MDCs secreted bioactive IL-12, a critical factor in switching naive or memory T cells to Th1 response[23]. In agreement with this result, the data presented here suggested that C44 and C48 induced low levels of proinflammatory cytokines (TNF-α, IL-6 and IL-8), while inducing high levels of IFN-γ and IL-12p70 but not IL-10, the formers are effective factors to promote cellular immunity and enhance the clearance of pathogens. Meanwhile, C44 is more suitable than C48 in the context of allergic diseases, based on its attenuated Th2 (IL-5, IL-6 and IL-10) potential. Th1 responses, as reflected by IFN-γ and IL-12p70 production, were strongly induced by C44 in anti-CD3 antibody activated PBMCs[12]. These results suggested that C44 and C48, especially C44, may be of potential application in allergic diseases.

It is reported that lactobacilli activate innate immune cells such as APCs via pattern recognition receptors (PRRs) and induce the secretion of cytokines that influence the polarization of activated T cells[9],[10] instead of interacting with lymphocytes directly. Commensal microflora are not normally found in extra-intestinal sites such as mesenteric lymphoid nodules, spleen, liver or blood in mice. However, commensal bacteria are likely to be continuously traversing the mucosal epithelium at a very low rate and are processed by the host immune cells (DCs) associated with the gut. If the intact mucosal barrier is disrupted by inflammation or injury, indigenous bacteria can easily pass through the ulcerated areas of the mucosa, and perhaps even through loosened tight junctions[24]; as a result, immune competent cells have the chances to contact with commensal bacteria. In the food allergy mouse model, *L. casei* administration skewed the pattern of cytokine production by splenocytes toward Th1 dominance, and suppressed IgE and IgG1 secretion by splenocytes[3]. We presumed that as commensal bacteria, lactobacilli had regulatory effects usually under abnormal conditions when the immune system is activated such as allergy. Thus, different with non-activated PBMCs, anti-CD3 antibody activated PBMCs were utilized in vitro in our study, and monocytes were stimulated by lactobacilli. In other words, anti-CD3 activated PBMCs are ready models for screening the regulation on T cell polarization by lactobacilli.

The components of probiotics that are responsible for modulation of cytokine induction are largely not known but might be involved in modification of microbe associated molecular patterns (MAMPs) such as lipoteichoic acids (LTA) and (lipo) proteins localized on the bacterial cell surface and interacting with toll like receptors (TLRs), especially TLR2 combining with TLR6[25]–[27]. Additionally, muramyl dipeptide (MDP), the degradation products of G+ bacteria cell wall, may interact with other host pattern recognition receptors named nucleotide-binding oligomerization domain 2 (NOD2) in plasma of APCs[28]. These products by probiotic cells are the likely targets for strain-dependent interactions with host cells and have been the focuses of several recent reviews[29]–[31]. In the present study, the effects of viable and heat-killed lactobacilli on cytokine production were not the same. As reported, viable lactobacilli may contact with monocytes in PBMCs via components on complete cell wall including LTA and peptidoglycan (PGN). Although most heat-killed lactobacilli had integral cell wall, degradation was inevitable; thus, MDP may be produced. These components binding different PRRs resulted in different signal transductions. This may explain the analogical effect of viable and heat-killed lactobacilli.

Unlike viable and heat-killed lactobacilli, genomic CpG DNA of lactobacilli led to mild inflammation via inducing lower levels of proinflammatory cytokines (IL-6, IL-8 and TNF-α) and cellular immunity (IL-12 and IFN-γ). The results suggest that DNA of lactobacilli is not suitable for the prevention and treatment of allergic disease compared with viable and heat-killed lactobacilli. This behavior may be caused by different receptors. DNA of lactobacilli induced cytokines in a TLR9-dependent manner that may lead to different signal transductions induced by complete and degradative components of the cell wall[32].

In conclusion, the lactobacilli isolated here have the probiotic characteristics not only of microbiological properties but also immuno-regulation by enhancing cellular immunity and reducing Th2 differentiation in vitro. Anti-CD3 antibody activated PBMCs is an effective model that allows pre-selection of probiotics to modulate the host immune system in vitro while reducing considerably the use of animals for screening purposes. Besides, candidate lactobacilli need to be assessed in animal tests and clinical trials for the prevention and treatment of allergic diseases.
